# Genomic analysis and in vivo efficacy of *Pediococcus acidilactici* as a potential probiotic to prevent hyperglycemia, hypercholesterolemia and gastrointestinal infections

**DOI:** 10.1038/s41598-022-24791-5

**Published:** 2022-11-28

**Authors:** Hassan M. Al-Emran, Jannatul Ferdouse Moon, Md. Liton Miah, Nigar Sultana Meghla, Rine Christopher Reuben, Mohammad Jashim Uddin, Habiba Ibnat, Shovon Lal Sarkar, Pravas Chandra Roy, M. Shaminur Rahman, A. S. M. Rubayet Ul Alam, Ovinu Kibria Islam, Iqbal Kabir Jahid

**Affiliations:** 1 Department Biomedical Engineering, Faculty of Engineering, Jashore University of Science and Technology, Jashore, Bangladesh; 2 Department of Microbiology, Faculty of Biological Sciences and Technology, Jashore University of Science and Technology, Jashore, Bangladesh; 3 German Centre for Integrative Biodiversity Research (iDiv), Halle-Jena-Leipzig, Germany; 4 Department of Pharmacy, Faculty of Biological Sciences and Technology, Jashore University of Science and Technology, Jashore, Bangladesh

**Keywords:** Bacteria, Immunology

## Abstract

Lactic acid bacteria are the well acknowledged probiotics that can cure a variety of diseases. In this study, we observed the in vivo potentials of *Pediococcus* to treat hyperglycemia, hypercholesterolemia and gastrointestinal infections. A total of 77 *Lactobacillus* were isolated from the milk of 10 cows and 10 goats, four of those strains inhibited both carbohydrates-hydrolyzing enzymes, α-glucosidase, and α-amylase. They all showed antagonistic effects on pathogenic *E. coli* and *S.* Typhimurium which were confirmed by performing pathogen challenge test and visualizing on Electron microscopy. 16S rRNA gene sequence identified that all four strains belong to *Pediococcu*s genus which were further distinguished as *Pediococcus acidilactici* by *phe*S gene sequence. Whole genome sequence analysis revealed their non-pathogenic properties for human and the presence of probiotic genes responsible for stress resistance, immunomodulation, adhesion, metal and drug resistance. In vivo trial with diabetes-induced mice ascertained that all *Pediococcus acidilactici* had significant potentials to reduce elevated glucose and low-density lipoprotein level in blood. Interestingly, two out of four strains were significantly more effective (p < 0.0001 each) than metformin in reducing the blood glucose level. This in vivo study demonstrated that *Pediococcus acidilactici* might be a promising probiotic to prevent hyperglycemia, hypercholesterolemia and gastrointestinal infections.

## Introduction

Diabetes mellitus is a life-threatening non-communicable metabolic disorder that accounts for 4.2 million adults and 1.1 million juvenile deaths in 2019^1^. Globally 463 million adults are infected with this non-infectious disease of which 232 million people are unaware that they have diabetes^1^. Nevertheless, this is the most frequent cause for seeking health care in low- and middle-income countries. Management of those large numbers of patients costs almost 827 billion US$ every year^2,3^. However, only 4% to 66% of the total diabetes patients take medicine to reduce their blood glucose level^4^. The primary explanation behind this reality may be the limited access to metformin (27%) or insulin (63%) in terms of availability and affordability in low-income countries^1^. There is enormous research ongoing to uncover the potency of antidiabetic chemicals, protein extracts from plants and bacteria to reduce blood glucose level^5–7^. Inhibition of the carbohydrates-hydrolyzing enzymes, α-glucosidase, and α-amylase is the most potential target to reduce the postprandial blood glucose level. However, most of the chemical compounds and protein extracts are partially effective and have side effects that make it unrealistic to treat diabetes^7,8^. There are a limited number of studies that utilize probiotic isolates to inhibit both of those enzymes^6,9,10^. Probiotics are “live microorganisms that, when administered in adequate amounts, confer a health benefit for the host”^11^. The first criteria for selecting a probiotic strain is that it has to be non-pathogenic for the host and it should not be responsible for horizontal resistance gene transfer to other flora^12^. In the last 5 years, a large number of original research has been published on “lactic acid bacteria AND probiotics” (4951 found in pubmed until 15th October, 2022). Therefore, the scientific interest of using this microorganism has increased exponentially to prevent and treat various diseases including bowel syndrome, hyperglycemia, hypercholesterolemia, neurological disorder etc.^13–16^. Use of lactic acid bacteria to treat gastrointestinal disorder^14,17^ has also been well acknowledged with no known adverse effect. *Salmonella, Campylobacter, E. coli* and *Listeria* are the common pathogens of concerns in animal farming, while Lactic acid bacteria can prevent a number of bacterial pathogens^18,19^. Lactobacillus secrets different organic substances like bacteriocins and antimicrobial peptides that inhibit the cell wall synthesis of other bacteria with no harmful effects on human^20^. *Pediococcus* are one of the members of the *lactobacillaceae* family and are already in use as probiotics in the production of sausage, yogurt and cheese. They are also used in silage and fed to cattle, sheep and other ruminants. Although very few studies have been published on *Pediococcus acidilactici* related with cholesterol and gastrointestinal disorder^21^ and none of them are on blood glucose. Our primary focus in this study was to acquire prospective lactobacillus strains that have the potentiality to prevent hyperglycemia and hypercholesterolaemia without any harmful effects in diabetes induced mice.

## Results

### Isolation and characterization

This study identified a total of 77 lactic acid bacterial isolates from 20 different milk samples. The preliminary identification was interpreted based on their negative test results in indole, MR, VP, citrate, catalase and oxidase. Only 12 (16%) out of 77 isolates showed inhibition of α-glucosidase and 7 (11%) showed inhibition of α-amylase. Only 4 isolates (ID: C3, C6, G11 and G13) have the ability to inhibit both enzymes, thus the rest 73 were excluded from this study. Those 4 strains were able to grow (O.D. > 0.500) in 6.5% NaCl concentration compared to blank (O.D. < 0.020). In 0.4% phenol solution, the indicator of growth in terms of O.D. were 0.350, 0.490, 1.01 and 1.05 representing their degree of tolerance. All strains were able to resist 0.3% bile salt concentration by growing on MRS agar plate after overnight incubation (Table 1). Their antagonistic abilities were low to moderate, producing zone diameter ranges between 14 and 21 mm against *E. coli* O157:H7 (ATCC 43894). The antagonistic ability against *Salmonella enterica* serovar Typhimurium (*S.* Typhimurium) (ATCC 14028) were ranged between 14 and 17 mm and against *E. faecalis* (ATCC 51299) were 15 to 17 mm. All isolate showed non-hemolytic character after inoculating on blood agar plates. Antibiogram test suggested that all probiotic isolates were sensitive to penicillin, amoxicillin and chloramphenicol. Two out of four strains were resistant to tetracycline and one was resistant against erythromycin (Table 1).

**Table 1:** Properties of the probiotic isolates.

Isolate ID	C3	C6	G11	G13
Source	Cow milk	Cow milk	Goat milk	Goat milk
α-amylase inhibition (%, average)	33	41	36	31
α-glucosidase inhibition (%, average)	23	17	30	21
6% NaCl tolerance test (OD_600nm_)	Yes ()	Yes	Yes	Yes
0.3% Bile salt tolerance test	Growth positive	Growth positive	Growth positive	Growth positive
0.4% Phenol tolerance test (OD_620nm_)	Yes (0.350)	Yes (0.490)	Yes (1.01)	Yes (1.05)
Antagonistic ability against *E. coli* O157:H (zone diameter in mm)	Yes (21)	Yes (19)	Yes (14)	Yes (16)
Antagonistic ability against *S.* Typhimurium (zone diameter in mm)	Yes (17)	Yes (16)	Yes (14)	Yes (14)
Antagonistic ability against *E. faecalis* (zone diameter in mm)	Yes (17)	Yes (16)	Yes (15)	Yes (17)
Susceptible against Antibiotics*	PEN, AMX, CHL	PEN, AMX, TET, CHL, ERY	PEN, AMX, TET, CHL, ERY	PEN, AMX, CHL, ERY
Non-susceptible against Antibiotics*	TET, ERY	–	–	TET
Gastric juice survivability	Yes	Yes	Yes	Yes
Ability to grow at 48 °C	Yes	No	Yes	Yes
16s RNA Identification with pheS gene and mutation analysis	*Pediococcus acidilactici*	*P. acidilactici*	*P. acidilactici*	*P. acidilactici*
Accession no	MT269574	MT269575	MT269576	MT269577
Whole genome analysis	*P. acidilactici*	*P. acidilactici*	*P. acidilactici*	Contamination
Accession no	JAFEMI000000000	JAFEMJ000000000	JAFEMK000000000	NA

*PEN* Penicillin, *AMX* Amoxicillin, *TET* Tetracycline, *CHL* Chloramphenicol, *ERY* Erythromycin.

*Cefoxitin, Ceftriaxone, Imipenum and Meropenum susceptibilities were not tested.

### Molecular identification and phylogeny

**Figure 1:**
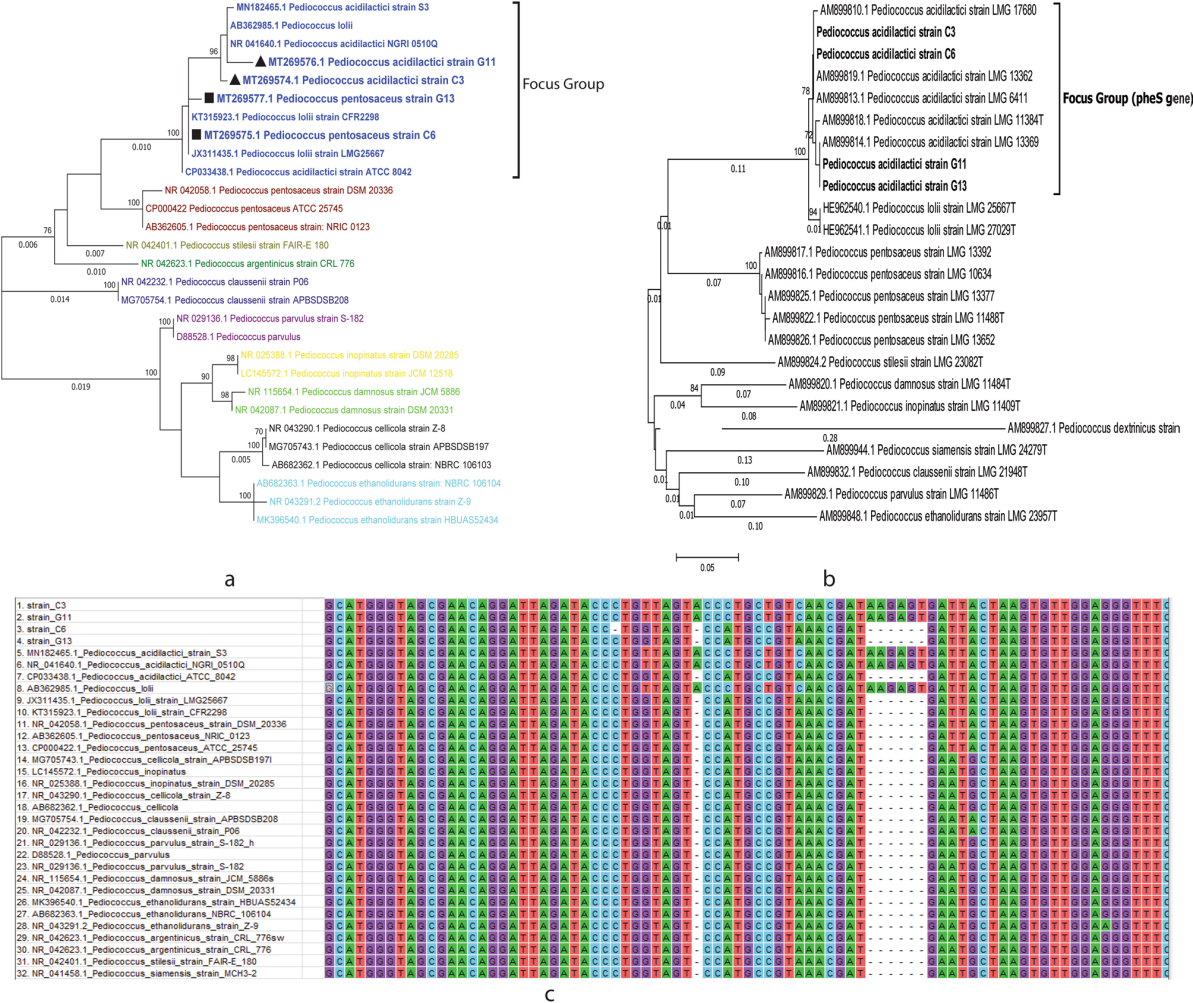
(**a**) 16S rDNA gene sequence based maximum likelihood tree represents the key grouping between *P. acidilactici* and *P. pentosaceaus* strains*.* However, due to very low diversity the phylogenetic tree could not differentiate between the *P. acidilactici* and *P. lolii*. (**b**) A *phe*S gene can differentiate between the *P. acidilactici* and *P. lolii* strains as shown in the focus group, and (**c**) The 6-bp deletion are found in the C6 and G13 strains that is not common in *P. acidilactici*. In both phylogenetics analyses (**a,b**), 1000 bootstraps were used and the trees showed > 70% values. The branch length values with more than 0.005 were shown in the tree.

The 16S rRNA gene sequence data confirmed that C3 and G11 strains were *P. acidilactici*. However, C6 and G13 laid in a different clade in the phylogenetic tree and it was hard to distinguish them between *P. acidilactici* and *Pediococcus lollii* (Fig. 1a). Mutational analysis from the 16S rRNA gene sequence data revealed a common deletion of 6 nucleotides (799 to 804 at C4 region) inside C6 and G13 strains (Fig. 1c). This deletion is commonly observed in *Pediococcus pentosaceus*, but not in *P. acidilactici*. Another distinguishing feature was the ability of *P. acidilactici* to grow at 48 °C, while *P. pentosaceus* couldn’t. In this study, C3, G11 and G13 grew on Mueller–Hinton Agar within 48 h at 48 °C and C6 didn’t grow even after 72 h. Then the *phe*S gene-based phylogeny interpreted all strains including C6 as *P. acidilactici* (Fig. 1b). All partial 16S sequence data of those isolates were deposited in the NCBI GenBank (Table 1).

### Genome analysis

**Figure 2:**
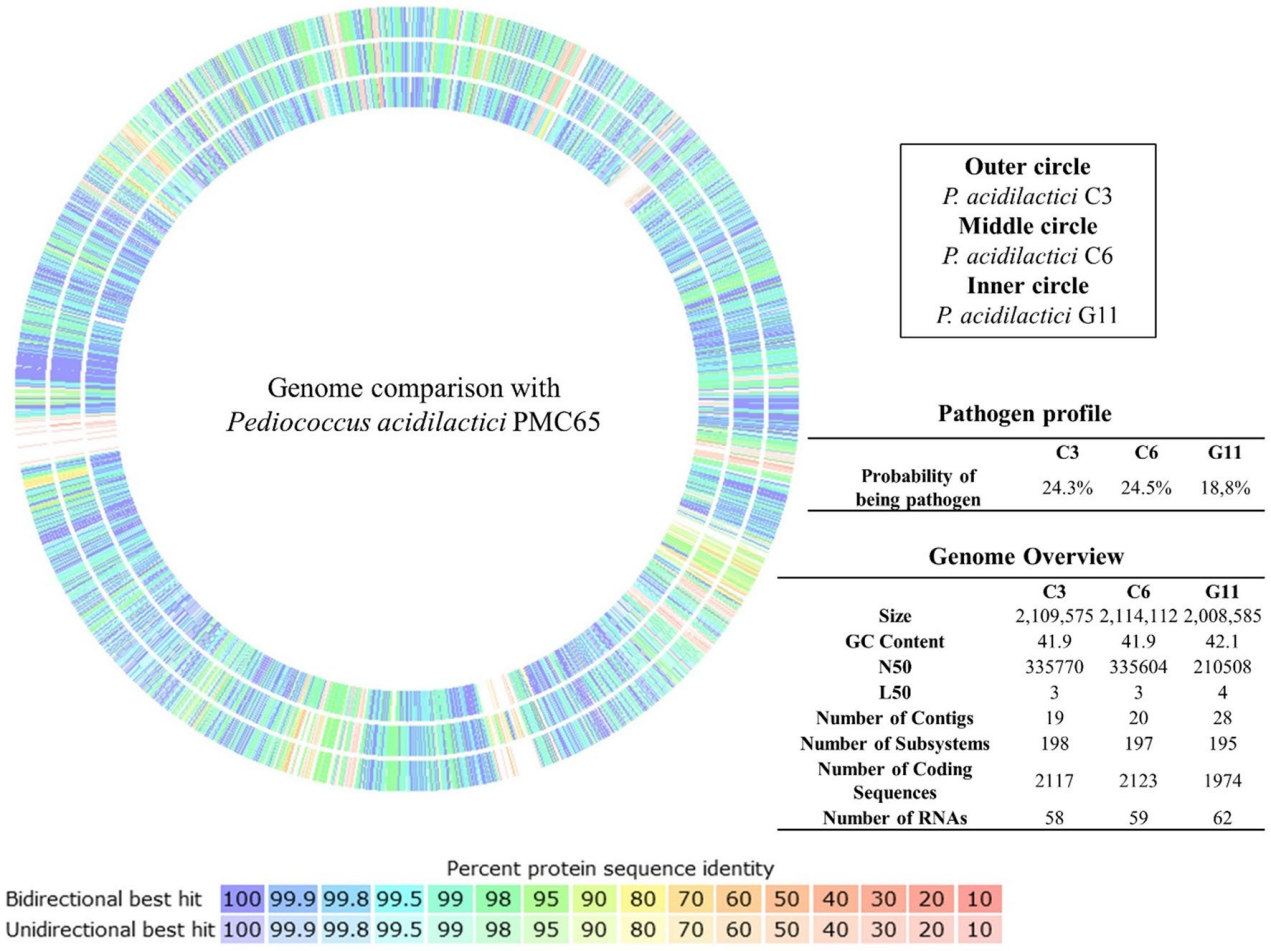
Genome content and Pathogen profiles of 3 *Pediococcus acidilactici* strains (C3, C6, G11). Genome comparison of C3, C6 and G11 strains showing protein sequence identities with reference strain PMC65 represented in circular view.

The whole genome analysis confirmed C3, G11 and C6 strains as *P. acidilactici*. G13 become contaminated during processing. The corresponding genome coverage, read length and GC content of C3 strains were 280x, 2.11 million bp and 41.9%, C6 strains were 284x, 2.12 million bp and 41.9%, and G11 strains were 429x, 2.01 million bp and 42.1%. They were assembled into 19, 20, 28 contigs and number of predicted protein coding CDS were 2117, 2123, 1974 respectively. PathogenFinder indicates that all 3 strains are nonpathogenic for human with 75.7%, 75.5% and 81.2% probability (Fig. 2). Additionally, no virulence gene/s were found in any isolate. C3 and C6 isolates sheltered tetM (tetracycline-resistant ribosomal protection protein) and ErmA (Erm 23S ribosomal RNA methyltransferase) antimicrobial resistance genes in addition to Glutathione biosynthesis bifunctional protein and 1188 aa thioredoxin reductase. Both strains showed 97% aa sequence homology of small multidrug resistance SMR-2 protein with SMR-2 from reference PMC65 strain. The presence of number of genes/proteins that are involved in stress resistance, active removal of stress, immunomodulation, adhesion, metal and drug resistance were found in all three genomes (Table 2).

**Table 2:** Presence of genes/proteins related to probiotic activity in *Pediococcus acidilactici* strain.

Name of the genes/proteins	Length (bp)	Function	Response	C3	C6	G11
**Stress resistance genes**						
*dlt*D	1287	d-anylation of LTA	Acid and defensin resistance	+	+	+
**DNA and protein protection and repair**						
*clp*C	2472	clpATPase	Persistence capacity in vitro	+	+	+
*msr*B	453	Methionine sulfoxide reductase	Persistence capacity in vitro	+	+	+
**Immunomodulation**						
*dlt*B	1206	d-anylation of LTA	Anti inflammatory potential in vitro in PBMC	+	+	+
*dlt*D	1287	d-anylation of LTA	Resistance to beta-defensin-2	+	+	+
**Adhesion ability**						
Fibronectin/fibrinogen binding protein	1716	Fibronectin/fibrinogen binding	Adhesion	+	+	+
**Active removal of stress**						
Cholylglycine hydrolase	1014	Bile salt hydrolysis	Tolerance to Bile salt	+	+	+
*gsh*F	1239	Glutathione biosynthesis bifunctional protein	Glutathione biosynthesis	+	+	−
Thioredoxin	312	Reduction by cysteine thiol-disulfide exchange	Tolerance to oxidative stress	+	+	+
Thioredoxin	321	Reduction by cysteine thiol-disulfide exchange	Tolerance to oxidative stress	+	+	+
Thioredoxin	330	Reduction by cysteine thiol-disulfide exchange	Tolerance to oxidative stress	+	+	+
Thioredoxin reductase	1188	Thioredoxin-disulfide reductase, pyrimidine conversions	Tolerance to oxidative stress	+	+	−
Thioredoxin reductase	984	Thioredoxin-disulfide reductase, pyrimidine conversions	Tolerance to oxidative stress	+	+	+
Thioredoxin reductase	924	Thioredoxin-disulfide reductase, pyrimidine conversions	Tolerance to oxidative stress	+	+	+
**Antibiotic resistance**						
Small multidrug resistance (SMR)-1	321	Small multidrug resistance	Drug resistance	+	+	+
Small multidrug resistance (SMR)-2	321	Small multidrug resistance	Drug resistance	97% identity	97% identity	+
Effux pump antibiotic resistance protein	405	Membrane bound transport	Drug resistance	+	+	+
Drug resistance tranporter, EmrB/QacA	729	Transport of drugs	Drug resistance	+	+	+
Multidrug resistance transporter, Bcr/CflA	1176	Transport of drugs	Drug resistance	+	+	+
Tet(M)	1920	Tetracycline resistance/ribosome protection	Drug resistance	+	+	−
**Metal resistance**						
CzcD	903	Cobalt/zinc/cadmium resistance	Metal resistance	+	+	+
Aluminum resistance protein	1260	Aluminum resistance	Metal resistance	+	+	+

### Field emission scanning electron microscopy

**Figure 3:**
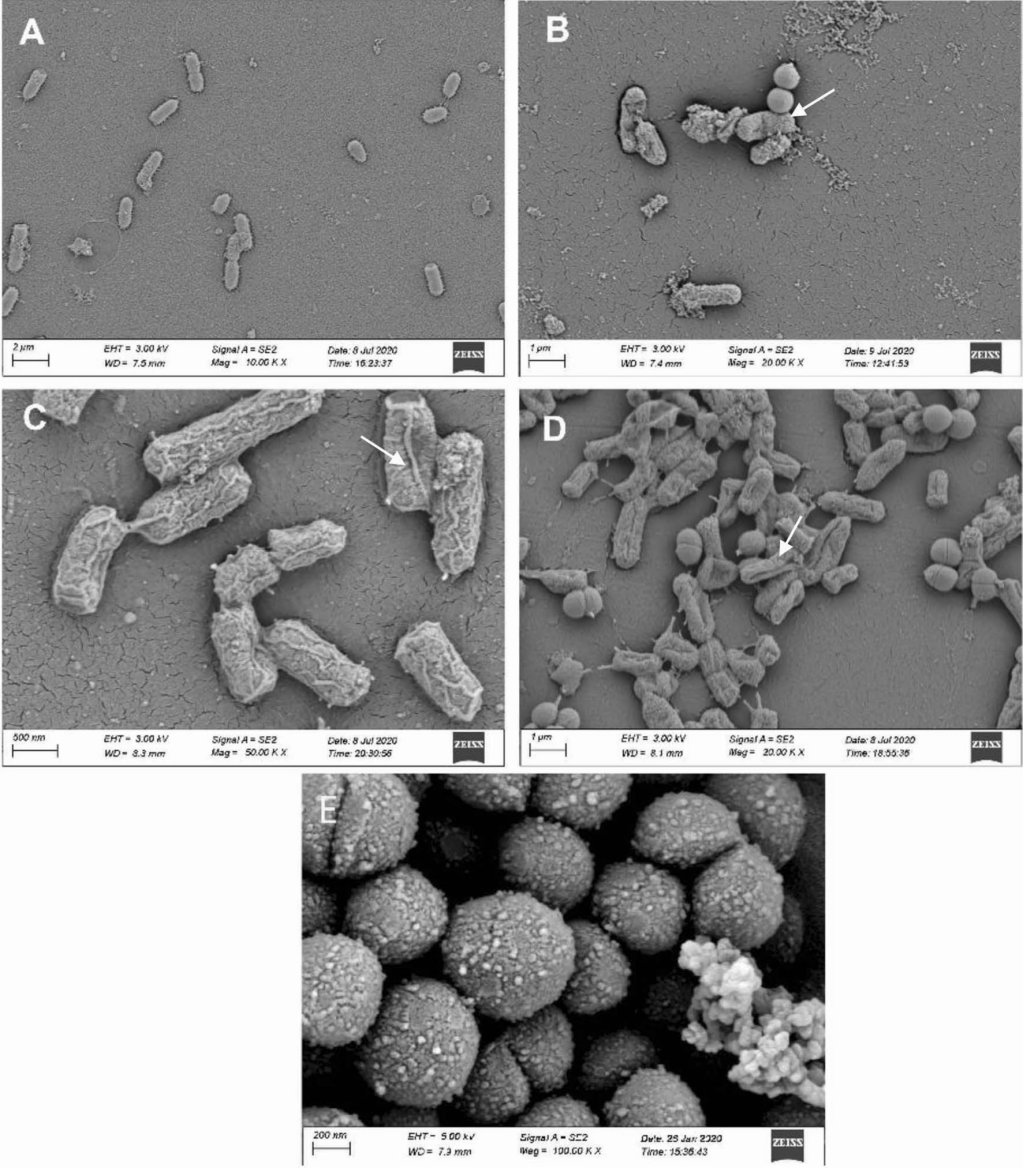
The Electron microscopy of *P. acidilactici* after 6 h of pathogen challenge in nutrient broth at 37 °C. (**A**) *E. coli* O157:H7 (ATCC 43894) grew independently as a control. (**B**) *E. coli* O157:H7 inoculated together with *P. acidilactici*. (**C**) *S.* Typhimurium (ATCC 14028) grew separately as a control. (**D**) *S.* Typhimurium (ATCC 14028) inoculated with *P. acidilactici*. (**E**) *P. acidilactici* grew independently without any pathogen challenge.

Electron microscope images represented the inhibition of *S.* Typhimurium and *E. coli* O157:H7 by *P. acidilactici* strains (Fig. 3). Figure 3B,D illustrated that all *S.* Typhimurium and *E. coli* O157:H7 cells were raptured and lysed completely by the antimicrobials secreted by *P. acidilactici.* This pathogen challenge test by electron microscopy further confirmed the capacity of our proposed probiotic strains to inhibit enteric pathogens.

### In vivo trial

#### Fasting blood glucose level

**Figure 4:**
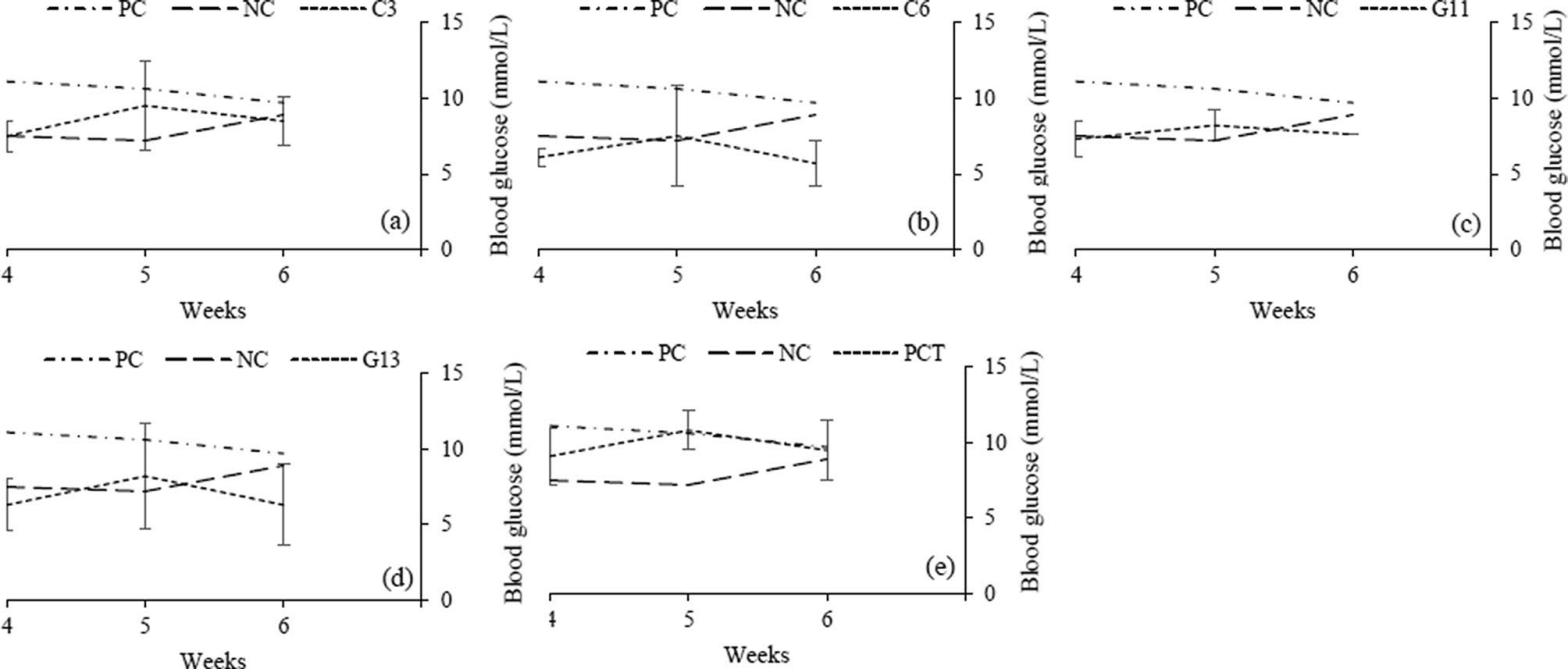
Graphical presentation blood glucose level (mmol/l) of positive control and negative control group compared with, (**a**) probiotic strain C3, (**b**) probiotic strain C6, (**c**) probiotic strain G11, (**d**) probiotic strain G13 and (**e**) Positive control treated with metformin (PCT).

The average fasting blood glucose readings (mmol/l) were 8.5(± 2) in C3, 6.6(± 2.2) in C6, 7.7(± 1) in G11 and 7(± 2.7) in G13 mice groups. However, the readings were 10.5(± 1.5) and 7.9(± 1.5) mmol/l in positive (PC) and negative control (NC) mice groups, respectively. One-way ANOVA significance test analysis indicates a significant drop of blood glucose in C6 (p < 0.00001), G11 (p = 0.012), G13 (p < 0.00001) and NC (p < 0.00001) group when compared with PC. The blood glucose levels of PCT (positive control treated with metformin) group, with an average of 9.8(± 1.8) mmol/l, were significantly higher than C6 (p < 0.00001) and G13 (p < 0.00001) groups (Fig. 4).

#### Lipid profile

The average lipid profile measured among all groups showed a significant decrease of low density lipoprotein (LDL) in all probiotic groups compared to positive control (PC) mice group and even negative control group (p = 0.015) according to the Duncan’s multiple range test (p > 0.05). However, HDL, TG and total cholesterol was also found to be decreased significantly compared to positive control with a p value of 0.006, < 0.001 and 0.001 respectively. G11 strains decreased the LDL (mean 26) into lowest keeping HDL high (mean 86) of all other strains (Table 3).

**Table 3:** Effect of probiotic strains supplementation on Lipid Profile of diabetic mice.

Parameter	C-3	C-6	G-11	G-13	PC	PCT	NC	SEM	P-value
HDL	73.00^bc^	52.00^c^	86.00^bc^	65.00^c^	144.00^a^	81.00^bc^	110.00^ab^	12.00	0.006
LDL	43.00^bc^	36.00^bc^	26.00^c^	34.00^bc^	89.00^a^	47.00^bc^	64.00^ab^	8.10	0.015
S. Chl	68.00^c^	53.00^c^	85.00^b^	63.50^c^	103.00^a^	60.00^c^	84.00^b^	6.60	0.001
TG	189.00^b^	179.50^bc^	128.00^d^	157.00^c^	247.00^a^	130.00^d^	192.00^b^	16.00	0.000

Values are the mean ± standard error of the mean of two replicates. Within each variable, values with the same superscript letter (^a, b, c, d, ab, bc^) are not significantly different according to Duncan’s multiple range test (P > 0.05).

#### Bodyweight

**Figure 5:**
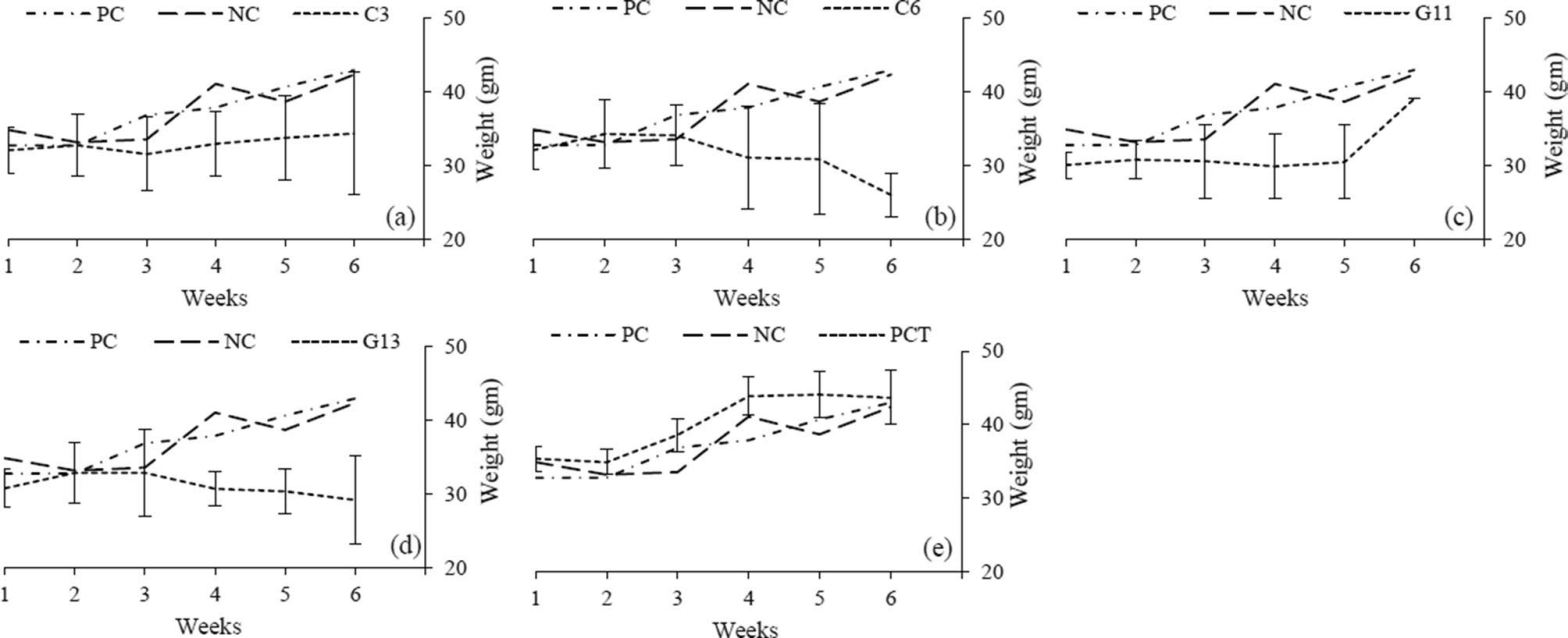
Body weight of the mice among different probiotic groups compared to positive and negative control groups. The vertical lines indicated the standard deviation from the mean value.

The average body weight (in g) was 33(± 4) in C3, 32(± 5) in C6, 30 (± 4) in G11 and 31(± 4) in G13 mice groups, compared to 37(± 6) in positive control (PC), 40(± 5) in positive control treated with metformin (PCT) and 37(± 5) in negative control (NC) mice groups (Fig. 5). One-way ANOVA significance test analysis depicted a significant drop in each probiotic group, when paired separately with control (PC, PCT and NC) mice groups (Table S1).

#### Survivability

In this study the survival rate of mice was 29% in C3, 43% in C6, 14% in G11, 71% in G13 and 86% in PCT groups, while no death was observed among positive (100% in PC) and negative (100% in NC) control groups (Fig. S1).

#### CRISPR defense system

C3, C6, and G11 harbored a complete type II CRISPR system with Cas9, Cas1, Cas2, and Csn2 proteins. The other locus of C3 and C6 contains 2 repeats of DR (Direct Repeat) length 47-bp and 24-bp sequence and spacer length 55-bp and 56-bp sequence without any Cas relevant genes. Additionally, G11 genome shelters 9 DR repeats where 2 locus contain Cas3 protein (Table S3).

#### KEGG and COG analysis

**Figure 6:**
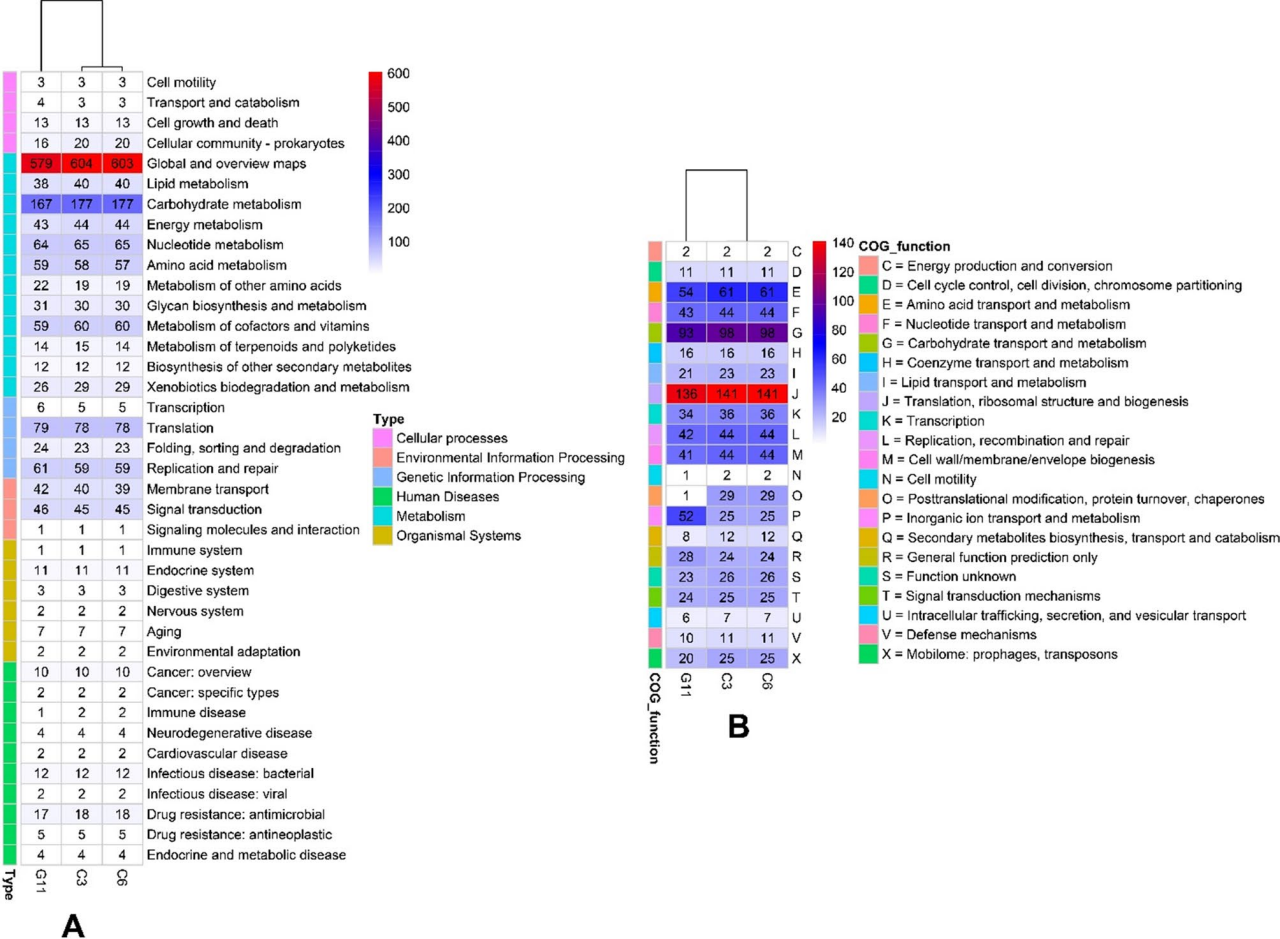
The number of genes assigned in KEGG (**A**) and COGs (**B**) categories. *KEGG* Kyoto Encyclopedia of Genes and Genome, *COGs* Clusters of Orthologous Groups.

KEGG pathway analysis revealed that a total of 1530, 1526 and 1494 genes are involved in 172, 171, and 177 KEGG pathways in C3, C6 and G11 respectively (Fig. 6A). The number of genes involved in different metabolism and processes have been studied. As shown in RAST, KEGG, and COGs also suggested that highest number of genes are involved in carbohydrate metabolism (Table 4). Total 706, 706 and 666 CDSs are divided into 21 functional categories in C3, C6 and G11 respectively using COG class description (Fig. 6B).

**Table 4:** Number of genes responsible for carbohydrate metabolism according to RAST and KEGG database.

Organisms	Carbohydrate metabolism (number of genes)		COGs
	RAST	KEGG	
C3	152	177	98
C6	153	177	98
G11	150	167	93

#### Bacteriocin

**Figure 7:**
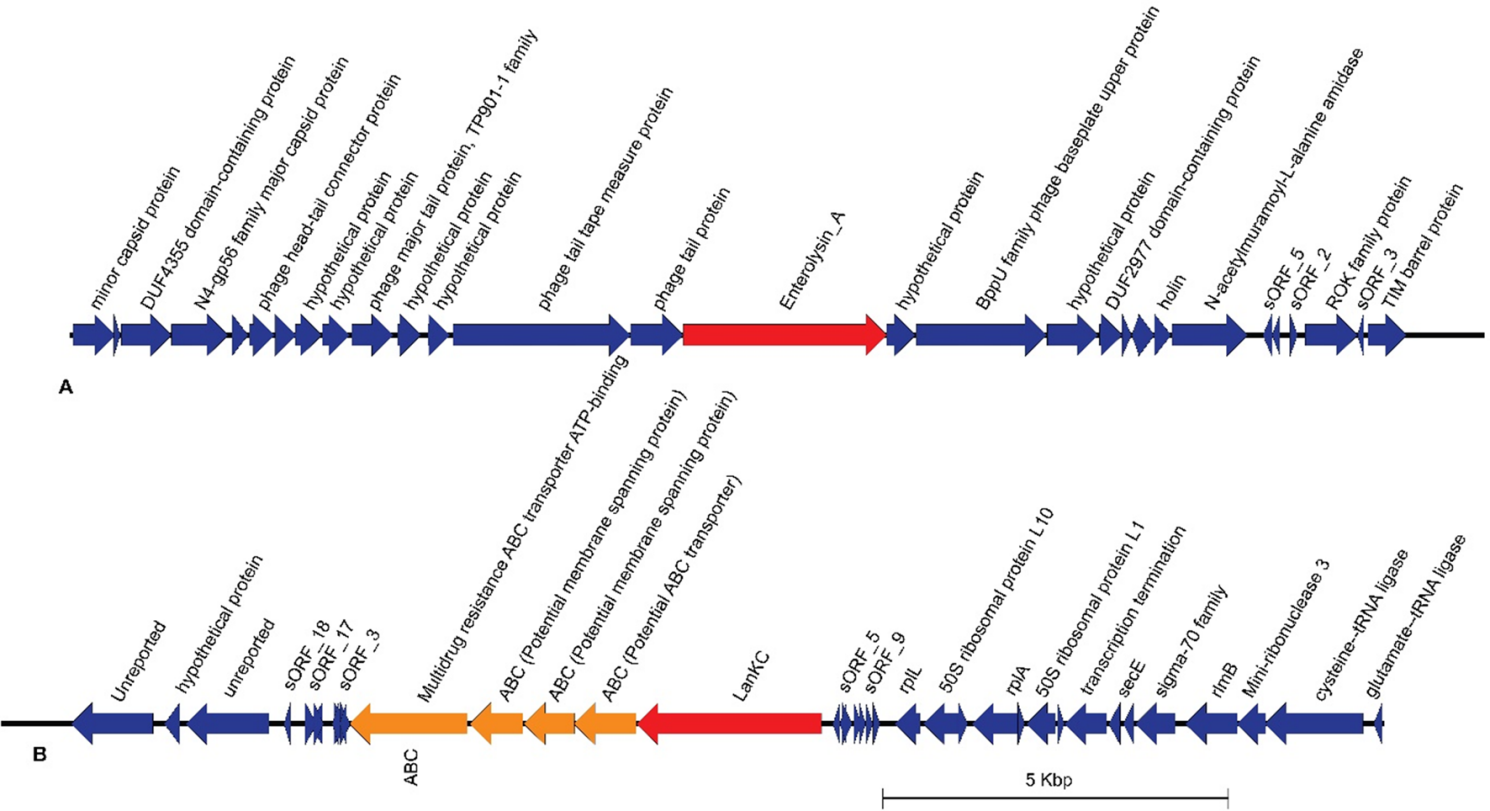
Putative area of interest (AOI) for the predicted bacteriocin in *Pediococcus acidilacti*. (**A**) C3 contains Enterolysin A class bacteriocin and (**B**) G11 contains Lanthipeptide class IV bacteriocin.

A bacteriocin-encoding loci were identified in the genome of C3 and G11 strains (Fig. 7). Enterolysin A class bacteriocin were observed in C3 genome. It was also surrounded by phage proteins, different genes, and hypothetical proteins. Strains G11 contains Lanthipeptide_class_IV bacteriocin. It was also surrounded by several other proteins such as ABC transporter, antibiotic resistance proteins, hypothetical proteins etc.

#### Carbohydrate metabolism

**Figure 8:**
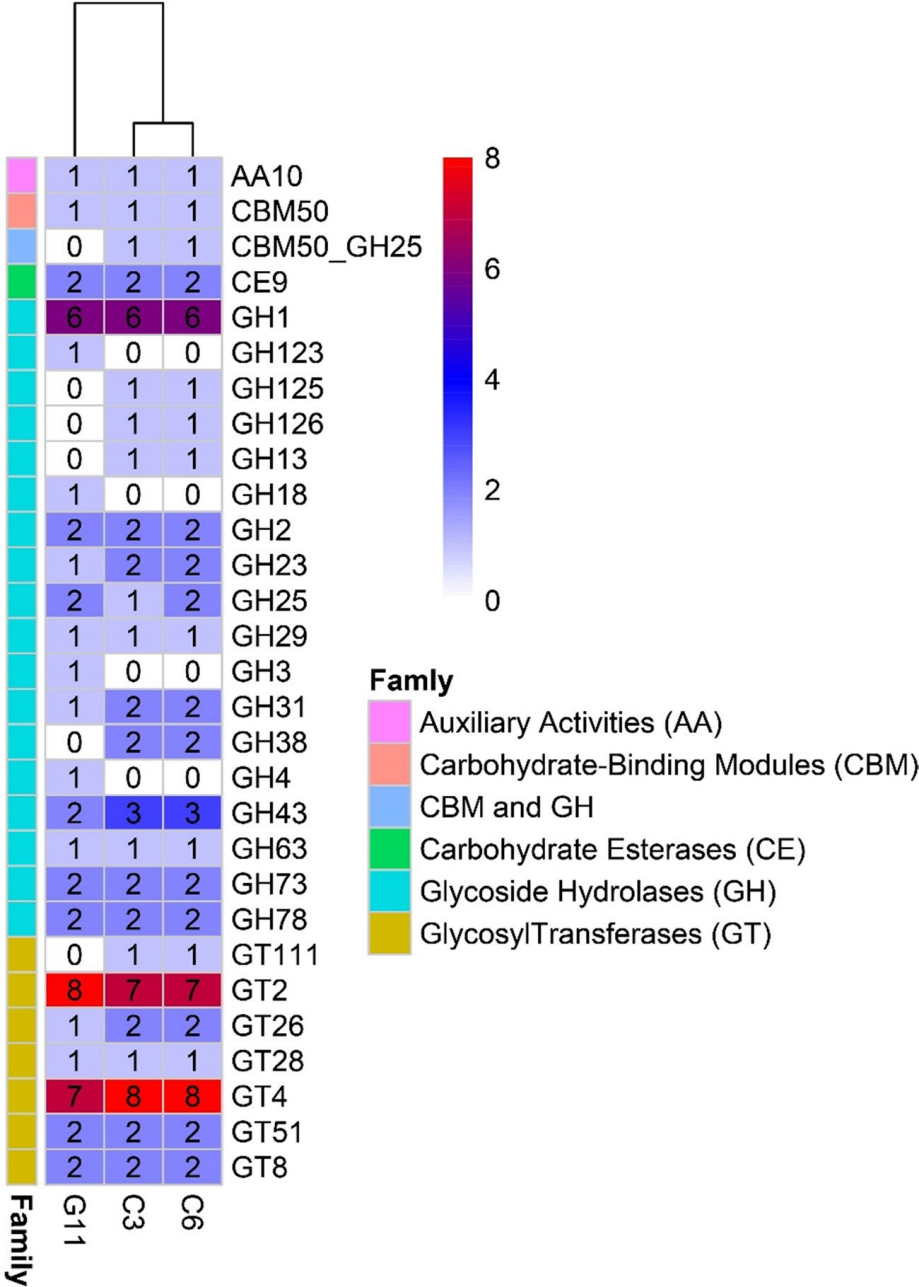
CAZymes present in C3, C6 and G11 were represented in a heatmap. Auxiliary Activities (AAs) represent redox enzymes that act in conjunction with CAZymes; Carbohydrate-Binding Modules (CBMs) represent adhesion to carbohydrates; Carbohydrate Esterases (CEs) represent hydrolysis of carbohydrate esters; Glycoside Hydrolases (GHs) represent hydrolysis and/or rearrangement of glycosidic bonds; and GlycosylTransferases (GTs) represent formation of glycosidic bonds.

The analysis of CAZymes revealed that the C3, C6 and G11 genomes contained 55, 56 and 49 genes respectively in the five CAZymes gene families. C3 and C6 possessed almost the same amount of CAZymes gene families: 1 auxiliary activity (AA) gene, 1 carbohydrate-binding modules (CBMs), 2 carbohydrate esterase (CE), 27 glycoside hydrolase (GH) genes (C6 contain 28 GH genes), and 23 glycosyl transferase (GT) genes. On the other hand, G11 sheltered 1 AA gene, 1 CBMs, 2 CE, 24 GH genes, and 21 GT genes (Fig. 8).

## Discussion

Diabetes mellitus is a state of chronic hyperglycemia due to the insufficient secretion of insulin that results an impaired uptake of blood glucose. It is the major risk factor for hypercholesterolemia and various other fatal diseases^1^. Dietary supervision and insulin secretion through medication can prevent or delay insulin independent diabetes^4^. This study isolated four *P. acidilactici* strains that have the capacity to reduce blood glucose in mice. Those strains showed in vivo*, *in vitro and in silico features to be used as a probiotic with the capacity to inhibit both α-amylase and α-glucosidase enzymes. Their non-hemolytic properties on the blood agar plate and whole genome sequence analysis using PathogenFinder also suggested that they were non-pathogenic for human.

The in vitro assessment of the study strains exhibited the capacity to withstand and grow within 6.5% NaCl containing broth. This is an important feature for a probiotic strain to tolerate variable range of salt concentration^22^. Phenol tolerance and intestinal bile salt tolerance both are key features for a probiotic strain to survive and grow in the gastrointestinal tract^23,24^. Our strains exhibited an excellent capacity to grow significantly in presence of 0.4% phenol (p < 0.005) and 0.3% bile salts. Apart from those, potential probiotic strains should also have the capacity to tolerate the extreme acidic condition in the gut environment. The gastric juice (pH 2.0) is bactericidal for most exogenous microbes other than enteric bacteria while ingested into the gastrointestinal tract. Our study strains showed the capacity to survive and grow within gastric juice containing MRS broth. All those in vitro physicochemical tests elucidated that any of these study stains would functionally inhibit both α-amylase and α-glucosidase enzymes and successfully reduce the blood glucose in the competitive environment of the gastrointestinal tract.

Antagonism is a consistent phenomenon in the common biological niche among different groups of bacteria. However, the degree of antagonism is strain specific. Two of our strains showed antagonistic effect with higher zone diameter than two others (Table 1). Electron microscopic observation in this study further confirmed the antagonistic effect of the study isolates on the reference enteric pathogens. Antibiotic susceptibility test results indicated that the probiotic strains were sensitive to most of the antibiotics (Table 1).

Our whole genome sequence analysis identified the characteristic probiotic genes responsible for stress resistance, active removal of stressors, bile salt hydrolysis, adhesion ability and immunomodulatory activity in all strains. They also possessed the genes for Choloylglycine hydrolase which play a vital role in bile salt hydrolysis^25,26^. Both *dlt*B and *dlt*D genes were present in all three strains, which are responsible for acid tolerance and anti-inflammatory potential^27^. Thioredoxin reductase is reported to be a key factor in oxidative stress response^28^. Three Thioredoxin reductases were present in C3 and C6, while G11 had only two. Multiple genes that are involved in antibiotic and metal resistance were also identified (Table 2). In the next phase of this study, we conducted a very successful in vivo trail for all 4 strains with 3 control groups and found that that the average blood glucose levels dropped significantly in 3 probiotic groups (p < 0.0001 in C6, p < 0.013 in G11 and p < 0.0001 G13) when compared with the positive control group (PC). Both C6 and G13 strain reduced blood glucose levels significantly better than metformin group (PCT) with p < 0.0001 each. The blood glucose level of the negative control group (NC) was significantly lower than the positive control group (p < 0.001) and metformin group (p < 0.013).

Abnormally low blood glucose can cause loss of consciousness and subsequently results in death. This may happen in all kinds of diabetes due to long fasting or over-exercise or high dose of medication. The low survivability rate (p < 0.00001 compared to PC) among the mice of probiotic groups clearly imposed the idea that the mice could have developed hypoglycemia when treated with probiotic strains. In that trial, 3 mice (one in C3, C6 and G11 group each) provided very low blood glucose levels (< 1.0, < 1.0 and 1.7 respectively) and subsequently each one died on the next day. Lactic acid bacteria are well established probiotics without any acknowledgment of causing diseases, and this remains the best proof of their safety^29,30^. These observations strengthen the study findings that the probiotic strains had the capacity of reducing the blood glucose level. Metformin has also a low risk of causing hypoglycemia^31^. The 14% fatality rate among the mice population of metformin groups (PCT) supports the same. Mice group treated with G13 strain also has higher survivability rate (71%). The reason for this may be the inoculum dose which is not optimized for individual strain. We used ca. 10^9^ cfu as a treatment dose followed by the method as depicted by Reuben et al.^32–34^. Treatment of diabetes mellitus with insulin or other medication helps to absorb glucose in our body that may result in weight gain^1^. In this study, we found a similar observation in the mice group treated with metformin. There was a significant gain of body weight among the metformin group (PCT) compared to the negative control group (p = 0.050). However, the probiotic strains didn’t show that effect. In all probiotic groups, there was significant loss of body weight when compared with positive control groups and at the same time with negative control group. In this study, the body average weights of the negative control group were continuously increased (Table 3).

Diabetes mellitus is the major risk factor for hypercholesterolemia. The death associated with diabetes is also due to this complication. The widely used medication, statins, has adverse side effects despite reducing circulating cholesterol in blood. This study represented from the lipid profile that there was a significant decrease of LDL (p = 0.015), triglyceride (p < 0.001) and total cholesterol (p = 0.001) in all probiotic mice groups compared to positive control and negative control mice group. LDL has also found to be significantly decreased in the metformin group (PCT) compared to PC group (Table 3). A recent study by Mitchel et al. 2017 isolated a probiotic consortium that decreased cholesterol level in mice blood^35^. This group recently reported a human trial that mentioned the *Lactobacillus* and *Bifidobacterium* consortium had the ability to reduce the elevated blood glucose and LDL level^9^. In another study, orally administered *P. acidilactici* in mice showed the capacity to reduce both blood glucose and serum triglyceride^21^.

Identification and confirmation of lactic acid bacteria is challenging because of their ambiguous response against biochemical tests. 16S rRNA sequencing approach is another way to identify them genetically at species level. However, this technique also sometimes failed to separate the species of *Pediococcus*^36,37^. This study confronted the similar situation during identifying the species of C6 strain. The use of protein-coding gene sequence data i.e., the alpha subunit of phenylalanyl-tRNA synthase (pheS), for the determination of genomic relatedness at the species level is suggested by Doi et al.^37^. Our 16S rRNA gene BLAST and phylogeny couldn’t initially specify two *Pediococcus* strains, C6 and G13 (Fig. 1). The common 6 nucleotide deletion (799 to 804 at C4 region) suggested by Doi et al.^37^ couldn’t assist for differentiating the strains in our study. The mutation analysis and temperature dependent growth criteria^36^ endorsed C6 strain as *P. pantosaceous* (Table 1). Although *pheS* gene and WGS analysis found all strains including the C6 as *P. acidilactici*, which indicated partial *pheS* gene analysis as suggested by Doi et al. can completely differentiate between *P. lolii* and *P. acidilactici*.

The presence of CRISPR loci may increase the genome stability of a bacterial strain, and therefore its adaptation in the environment^38,39^. All three strains in this study possessed complete type II CRISPR/Cas defense system like other *P. acidilactici* studies previously^40^. Commonly, *P*. *acidilactici* contain pediocin PA-1, enterolycin A and colicin-B^41^, although, we identified only enterolycin A for two strains (Fig. 6). Glycoside hydrolases (GH) are a widespread group of enzymes which hydrolyse the glycosidic bond between two or more carbohydrates or between a carbohydrate and a non-carbohydrate moiety^42^. The genome of C3, C6 and G11 contain the higher number of GH family genes. Glycoside transferase (GTs) catalyze the transfer of sugars from activated donor molecules to specific acceptors and are important for the formation of surface structures, which are recognized by host immune systems^43^. All three strains carried almost close number of GTs genes of GHs family (Table S2).

Our study revealed that all four probiotic strains had in vitro ability to inhibit α-amylase and α-glucosidase enzyme as well as enteric pathogens (Fig. 1). In vivo trial revealed that three *P. acidilactici* (C6, G11 and G13) have significant potential to reduce elevated glucose and LDL level in mice blood. C6 and G13 strains were significantly more effective than metformin (p < 0.0001 each) in reducing the blood glucose level and body weight. The whole genome analysis of those strains revealed that they were non-pathogenic for human, possessed the necessary genes to inhibit pathogens, attained important probiotic featured genes like bacteriocin, resistance to bile salt and gastric juice, adhesion properties, and for active removal of stressful or competitive environments. The in vitro*, *in vivo and in silico analysis of this study found that *P. acidilactici* has the potentials to serve as probiotics by preventing hyperglycemia, hypercholesterolemia and gastrointestinal infections. Recent studies also suggested the use of probiotics to treat various diseases as an alternative to conventional medication. Considering the fact that the chemical medications have substantial side effects and resistances, the study outcomes may contribute to next generation treatment policies.

## Methods

### Sample collection

Milk samples were collected from 10 red Chittagong cows and 10 Black Bengal goats resided in 3 different communities Abdulpur (23.2244° N, 89.1453° E), Shamnagar (23.1439° N, 89.0732° E) and Ambottola (23.230925° N, 89.129355° E) in Jashore, Bangladesh. All samples were collected aseptically in a 100 ml sterile container (Duran, USA) and transported to the laboratory immediately by maintaining a cold chain.

### Isolation of lactic acid bacteria

The collected milk samples (1 ml) were added to 10 ml of MRS broth (Sigma-Aldrich, Germany) and incubated at 37 °C for 48 h^44^. All samples were plated on MRS agar (Sigma-Aldrich, Germany) and further incubated at 37 °C upto 72 h until observed bacterial growth. The colonies were preliminary identified with the Gram staining, cell morphology, indole, MR, VP, citrate, catalase test and coagulase test in standard procedure (Sharpe 1979)^32,45^.

### Α-Amylase inhibition assay

α-Amylase inhibition assays were performed by the method described in Worthington Enzyme manual (1993)^46^ with a minor modification. Briefly, bacterial culture was suspended in 500 µl of PBS and mixed with 500 µl of sodium phosphate buffer (pH 6.9) containing a-amylase solution (0.5 mg/ml) (Sigma-Aldrich, Germany) and incubated at 25 °C for 10 min. Another 500 µl of starch solution (1% in sodium phosphate buffer) was added to each tube and incubated again at 25 °C for 10 min. The reaction was stopped with 1.0 ml of dinitrosalicylic acid (Sigma-Aldrich, Germany) color reagent. The test tubes were placed in boiling water for 5 min and cooled to room temperature. 5 mL of distilled water was added to the mixture, and the absorbance was measured at 540 nm using the spectrophotometer (PG instrument, UK).

### Α-Glucosidase inhibition assay

α-Glucosidase inhibition assays were performed using the technique described by Zheng et al.^47^. In brief, bacterial culture was suspended in 50 µl of PBS and incubated at 37 °C for 10 min after mixing with 25 µl of *p*-nitrophenol α d-glucopyranoside (10 mM) and 25 µl of PBS (pH 6.8). α-glucosidase (1 U/ml) (Sigma-Aldrich, Germany) were added of 50 µl volume and incubated at 37 °C for 30 min. The reaction was stopped by adding 100 μl of Na_2_CO_3_ (0.1 M), and the absorbance of the samples were measured at 450 nm in a microplate Reader (ALTA ELISA reader, Athenese-DX, USA).

### Tolerance tests

NaCl tolerance tests were performed at 1% to 6.5% increasing concentration of NaCl in MRS broth^48^ and bile salt tolerant tests were performed at 0.3% concentration of bile salts (Marek, Germany) in MRS broth^32^. Phenol tolerance tests were performed at 0.1% and 0.4% increasing concentration of phenol dissolved in MRS broth as described by Reuben et al. 2019^44^. Gastric juice survivability tests were also performed using the technique as described in the same study. Antagonistic tests against *Escherichia coli* O157:H7 (ATCC 43894), *Salmonella* Typhimurium (ATCC 14028) and *Enterococcus faecalis* (ATCC 51299) were performed using agar well diffusion assay described in Prabhurajeshwar et al.^48^. Hemolytic activity has been tested for all isolates by inoculating the strains on blood agar medium containing 5% sheep blood. The colonies have been observed after overnight incubation at 37 °C. Antibiotic susceptibility tests have been performed by disc diffusion method against Penicillin, Amoxicillin, Tetracycline, Chloramphenicol and Erythromycin, and interpreted according to the CLSI guidelines (CLSI 2015). Cefoxitin, Ceftriaxone, Imipenum and Meropenum susceptibilities were not tested.

### Field emission scanning electron microscopy

Antagonistic effects were confirmed in scanning electron microscope (Ziess SEM). Freshly prepared culture of *E. coli* O157:H7 (ATCC 43894) and *S.* Typhimurium (ATCC 14028) were mixed separately with selected strains in nutrient broth and incubate at 37 °C for 6 h. The test was repeated with both C6 and G11 strains. The bacterial cells were fixed on plastic surfaces and treated as describe by Jahid et al.^49^. The dehydrated samples were sputter coated with gold particles, visualized and photographed by field emission scanning electron microscope (FESEM) (Sigma 300, Carl Zeiss, Germany).

### In vivo trial with probiotic strain

Swiss Webster male mice were collected from the Ethnopharmacology laboratory of Jahangirnagar University, Bangladesh. Each mouse weighed within the range of 30 to 35 g and kept in a well-ventilated animal house for one week prior to in vivo trial. A total of 49 mice were distributed into 7 groups (7 mice each) in this trial; four groups were for probiotic strains (C3, C6, G11 and G13) as a proposed treatment, one group was for positive control that received oral metformin (300 mg/kg) as a commonly used treatment regime (PCT), another was positive control group (PC) that did not received any treatment and one was for negative control group (NC) that had neither induced diabetes nor received any treatment. Four selected probiotic strains (ca. 10^9^ cfu) were suspended in 250 µl skim milk^50^ and. ingested through oral gavages in four mice groups once in everyday upto 6 weeks. Three other control groups received only 250 µl skim milk instead. After the third week, diabetes was induced with a single dose of Streptozotocin (40 mg/kg) in all mice except the mice of the negative control group following a previous study with a minor modification^50^. The dose was reduced to half (instead of 80 mg/kg) by our preoptimization study (data not shown). Fasting blood glucose level was measured early in the morning after the 4th week, 5th week and 6th week with the portable glucose meter (Glucoleader™, Taiwan). Body weight was measured (Model: XB 220A SCS, Precisa, Switzerland) every first day of each week. Lipid profile is measured in Siemens Dimension RxL/Max/Vitros350 Random Access Chemistry Analyzer (Siemens Healthcare Diagnostics Inc, USA) according to the manufacturer instructions. Serum samples were obtained by sacrificing 2 mice of each group at 7th week. The mean and standard deviation of body weight, blood glucose level and lipid profile was calculated separately for each group.

### Molecular identification of the isolates

The entire 16S ribosomal RNA gene were amplified by widely used primer set (27F: AGAGTTTGATCMTGGCTCAG and 1492R: TACGGCTACCTTGTTACGACTT) targeting ca. 1500 base pair (weisburg et al. 1991). PCR was performed in a total volume of 20 μl including 10 μl of 2× master-mix, 0.5 μl of both primers, 2 μl template DNA extract and 7 μl of nuclease-free water^23^. The PCR products were purified using the Promega PCR purification kit (Promega, WI, USA). The amplicons were mixed with The BigDye Terminator v3.1 cycle sequencing kit and sequenced in ABI genetic analyser (Applied Biosystems, USA). The sequence data were compared using the basic local alignment search tool (BLAST) for the initial identification of the strains. A maximum likelihood (ML) model based phylogenetic tree was generated and mutation analysis was performed at the DNA sequence level using MEGA7 to find more accurate variation among those bacterial isolates (Wieme et al.). Additionally, we used pheS-21-F (CAYCCNGCHCGYGAYATGC) and pheS-22-R (CCWARVCCRAARGCAAARCC) primer pair to amplify a ~ 470 bp amplified product for further identification at species level^51^. Neighbour-joining tree was generated in MEGA7 for pheS gene where the Tamura-3 parameter model was used.

### Bacterial whole genome sequencing and assembly

DNA from the pure culture were extracted using QIAamp DNA Mini Kit (Qiagen, Hilden, Germany) according to the manufacturer’s instructions. The quality and quantity of the extracted genomic DNA were assured by Nanodrop ND-200 (Thermo Fisher, Waltham, MA, USA) and the integrity was assured by agarose gel electrophoresis.

Genomic DNA libraries were constructed using Nextera XT DNA Library Preparation Kit (Illumina Inc., San Diego, USA). The WGS was performed using 151 bp paired-end sequencing protocol under Illumina platform using HiSeq4000 sequencer according to the manufacturer’s instruction (Macrogen, lnc. Seoul, Republic of Korea). In brief, the library was prepared by random fragmentation followed by adaptor ligation. Adaptor ligated fragments were amplified by PCR and purified. The library was then loaded into a flow cell, captured on a surface bound oligos complementary to the adapters. Each single base was detected when it was incorporated into the DNA template. The Illumina sequencer generates raw images utilizing sequencing control software for system control and base calling through an integrated primary analysis software called RTA (Real Time Analysis). The BCL (base calls) binary is converted into FASTQ utilizing illumina package bcl2fastq (https://support.illumina.com/sequencing/sequencing_software/bcl2fastq-conversion-software.html) yielding an average ∼ 14 million reads per sample. The generated FASTQ files were evaluated for quality using FastQC v0.11^52^. Adapter sequences, and low-quality ends per read were trimmed by using Trimmomatic v0.39^53^ with set criteria of sliding window size 4; a minimum average quality score of 20; minimum read length of 50 bp and after trimming, an average 12.45 million reads per sample passed the quality checking steps. High quality reads were de novo assembled to generate contigs using an open source platform, SPAdes (Species Prediction and Diversity Estimation) v3.13^54^ with121 k-mer, and contigs less than 500 bp were filtered. Completeness and contamination of the genome contigs were checked using CheckM. Again, the genome contigs were searched for the bacterium at strain level by BLAST, and the k-mer algorithm in the KmerFinder 3.1 tool^55^.

### Genome annotation and analysis

The assembled draft genome of the isolate C3, C6 and G11 were annotated by using NCBI Prokaryotic Genome Annotation Pipeline (PGAPv4.11) with best-placed reference protein set, and GeneMarkS-2+ annotation methods^56^, Rapid Prokaryotic Genome Annotation (Prokka) (e = 0.000001)^57^. The genomes were also annotated by RAST (e = 0.000001) server^58^. The SEED viewer was used for the exploration and comparative analysis of annotated genes^59^. The nucleic acid and protein sequences of previously reported probiotic genes/proteins were retrieved from NCBI server. The nucleic acid and protein sequences were compared with the annotated genomes in the SEED viewer by BlastP analysis. Comparison of genomic profiles of C3, C6 and G11 was performed using the same server using *P. acidilactici* PMC65 as reference strain. Pathogenic profile of the sequenced genomes were also checked by PathogenFinder^60^ and VFDB (Virulence Factors of Pathogenic Bacteria)^61^, and drug resistance were checked CARD (Comprehensive Antibiotic Resistance Database)^62^.

### KEGG and COG analysis

For KEGG (Kyoto Encyclopedia of Genes and Genomes) annotation, KAAS–KEGG Automatic Annotation Serve (https://www.genome.jp/kegg/kaas/) and KEGG Mapper (https://www.genome.jp/kegg/mapper/) were used where annotated genome were used as an input. For KAAS, BBH (bi-directional best hit) for prokaryotic genes dataset were applied to complete or draft genome and KEGG Mapper—Reconstruct were applied to KEGG annotation^63^. For Clusters of Orthologous Groups (COGs) were assigned by Blast search with DIAMOND (ref) (E-Value < 1e−102, ≥ 80% identy and maximum target = 1) against NCBI COG database (updated 2020)^64^.

### CRISPR identification and characterization of isolated strains

The Clustered Regularly Interspaced Short Palindromic Repeats (CRISPR) regions and CRISPR-associated (Cas) proteins were identified by CRISPRCasFinder with default parameters^65^ (https://crisprcas.i2bc.paris-saclay.fr/CrisprCasFinder/Index) and the CRISPR subtypes designation was based on the signature of Cas proteins^66^.

### Bacteriocin identification

All of the strains were assessed for the presence of bacteriocin operons by BAGEL4 and the domains of bacteriocin were determined using BLASTP analysis against the non-redundant protein databases created by BLASTP based on NCBI^67^.

### Carbohydrate metabolism

The annotated genomes of the isolate C3, C6 and G11 were searched against CAZY database (http://www.cazy.org/) on dbCAN2 (http://bcb.unl.edu/dbCAN2/index.php)^68^ an online server by using DIAMOND^69^ blast search with E-Value < 1e−102 and ≥ 80% identity.

### Statistical analysis

All data were plotted in Microsoft Excel. The body weight and blood glucose data were analysed by one-way analysis of variance (ANOVA) test using SAS version 9.3 (SAS institute Inc, Cary, NC, USA). T-test were calculated with 95% CI by excluding the data outliers. Pooled variance T-tests were performed to compare between two variances.

### Ethical clearance

This study is approved the Ethical Review Committee of Jahangirnagar University and Jashore University of Science and Technology (ERC number: ERC/FBST/JUST/2019-31 date. 23.09.2019). The mice were kept under controlled environmental conditions and health status was monitored by expert veterinarian. Authors confirmed that all experiments were performed in accordance with relevant guidelines and regulations and in compliance with the ARRIVE guidelines.

## Supplementary Information

Supplementary Information.

## Data Availability

The whole genome sequence data analysed in this study were deposited in NCBI gene bank (https://www.ncbi.nlm.nih.gov/genbank/) with Accession No. of JAFEMI000000000, JAFEMJ000000000 and JAFEMK000000000 (Table 1).
