# Supplementary material for: Genomic analysis and in vivo efficacy of Pediococcus acidilactici as a potential probiotic to prevent hyperglycemia, hypercholesterolemia and gastrointestinal infections
Source: Sci Rep. 2022 Nov 28;12:20429. doi: 10.1038/s41598-022-24791-5 (PMC9705362; doi:10.1038/s41598-022-24791-5)
Supplement: Supplementary file 1 — Supplementary Information. [file 41598_2022_24791_MOESM1_ESM.docx]

**S1 Table:** p-value presented from the body weight of mice calculated using One-way ANOVA significance test.

| **Paired group** | **C3** | **C6** | **G11** | **G13** | **PC** | **PCT** |
| --- | --- | --- | --- | --- | --- | --- |
| **C3** |  |  |  |  |  |  |
| **C6** | 1.000 |  |  |  |  |  |
| **G11** | 0.822 | 0.948 |  |  |  |  |
| **G13** | 0.929 | 0.991 | 1.000 |  |  |  |
| **PC** | 0.002 | 0.000 | 0.000 | 0.000 |  |  |
| **PCT** | 0.000 | 0.000 | 0.000 | 0.000 | 0.185 |  |
| **NC** | 0.014 | 0.002 | 0.000 | 0.000 | 0.998 | 0.050 |

**S2 Table:** Number of gene family according to CAZymes database found on C3, C6 and G11 draft genome.

| Family | C3 | C6 | G11 |
| --- | --- | --- | --- |
| AA | 1 | 1 | 1 |
| CBM | 1 | 1 | 1 |
| CBM_GH | 1 | 1 | 0 |
| CE | 2 | 2 | 2 |
| GH | 27 | 28 | 24 |
| GT | 23 | 23 | 21 |

**S3 Table**: CRISPR defense system present in C3, C6 and G11 genome reveled by CRISPRCasFinder.

| ID | Contig (Start-End) | DR Consensus | DR Length (bp) | Spacers Count | Spacers Length (bp) | Conservation DR | Conservation Spacer | Cas_type |
| --- | --- | --- | --- | --- | --- | --- | --- | --- |
| C3 | NODE_1 (46441-46589) | TAGTCGAAACGGGTTTTGTAACCCAGCTCTCTCCGGCTAACCAAATT | 47 | 1 | 55 | 100 | 100 | - |
|  | NODE_3 (216552-217181) | GTTTCAGAAGGATGTTAAATCAATAAGGTTAAGATC | 36 | 9 | 30 | 97.39 | 0 | CAS-TypeIIA (Cas9_0_II, Cas1_0_II, Cas2_0_I-II-III, Csn2_0_IIA) |
|  | NODE_6 (0-0) | - | - | - | - | - | - | CAS-TypeVB (Cas4_0_V) |
|  | NODE_7 (135-238) | TTAGCTCAGTTGGGAGAGCATCTG | 24 | 1 | 56 | 95.83 | 100 | - |
| C6 | NODE_1 (451650-451753) | CAGATGCTCTACCAACTGAGCTAA | 24 | 1 | 56 | 95.83 | 100 | - |
|  | NODE_2 (316051-316199) | AATTTGGTTAGCCGGAGAGAGCTGGGTTACAAAACCCGTTTCGACTA | 47 | 1 | 55 | 100 | 100 | - |
|  | NODE_3 (216320-217015) | GTTTCAGAAGGATGTTAAATCAATAAGGTTAAGATC | 36 | 10 | 30 | 97.56 | 0 | CAS-TypeIIA (Cas9_0_II, Cas1_0_II, Cas2_0_I-II-III, Csn2_0_IIA) |
|  | NODE_6 (0-0) | - | - | - | - | - | - | CAS-TypeVB (Cas4_0_V) |
| G11 | NODE_19 (1-120) | TAATTCACCAACAACAGTTGACGAAGAGCC | 30 | 1 | 60 | 100 | 100 | - |
|  | NODE_2 (193626-194387) | GTTTCAGAAGGATGTTAAATCAATAAGGTTAAGATC | 36 | 11 | 30 | 87.36 | 0 | CAS-TypeIIA (Cas9_0_II, Cas1_0_II, Cas2_0_I-II-III, Csn2_0_IIA) |
|  | NODE_4 (1-121) | TTAATTCACCAACAACAGTTGACGAAGAGCC | 31 | 1 | 59 | 100 | 100 | - |
|  | NODE_6 (64198-64361) | TTAGACCTAAAATTTGGTTAACCGGAAAAAATTGGTGGCTGGAACGCGTTTCGGC | 55 | 1 | 54 | 98.18 | 100 | - |
|  | NODE_7 (1-127) | TTAATTCACCAACAACAGTTGACGAAGAGCCCGTTTT | 37 | 1 | 53 | 97.3 | 100 | - |
|  | NODE_7 (106150-106253) | CAGATGCTCTACCAACTGAGCTAA | 24 | 1 | 56 | 95.81 | 100 |  |
|  | NODE_8 (1-121) | TTAATTCACCAACAACAGTTGACGAAGAGCC | 31 | 1 | 59 | 100 | 100 | - |
|  | NODE_9 (71099-71218) | GGGCTCTTCGTCAACTGTTGTTGGTGAATT | 30 | 1 | 60 | 100 | 100 | - |
|  | NODE_12 (1-120) | TAATTCACCAACAACAGTTGACGAAGAGCC | 30 | 1 | 60 | 100 | 100 | Cas (Cas3_0_I, Cas3_0_I) |
|  | NODE_12 (51302-51422) | GGCTCTTCGTCAACTGTTGTTGGTGAATTAA | 31 | 1 | 59 | 100 | 100 | Cas (Cas3_0_I, Cas3_0_I) |

**
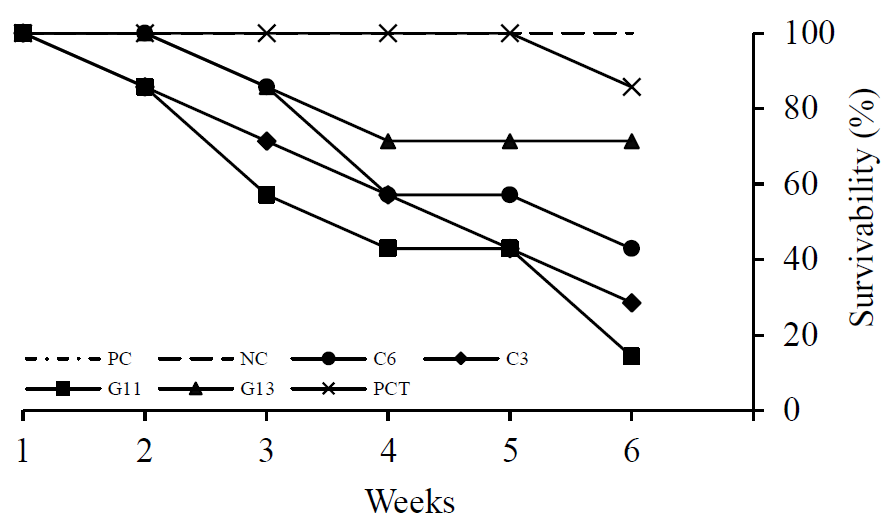
**

**S1 Fig:** Survivability curve of mice from different group.
